# Supplementary material for: Analysis of the Type 4 Effectome across the Genus Rickettsia
Source: Int J Mol Sci. 2022 Dec 8;23(24):15513. doi: 10.3390/ijms232415513 (PMC9779031; doi:10.3390/ijms232415513)
Supplement: Supplementary file 1 [file ijms-23-15513-s001.zip › Figure S1.pdf]

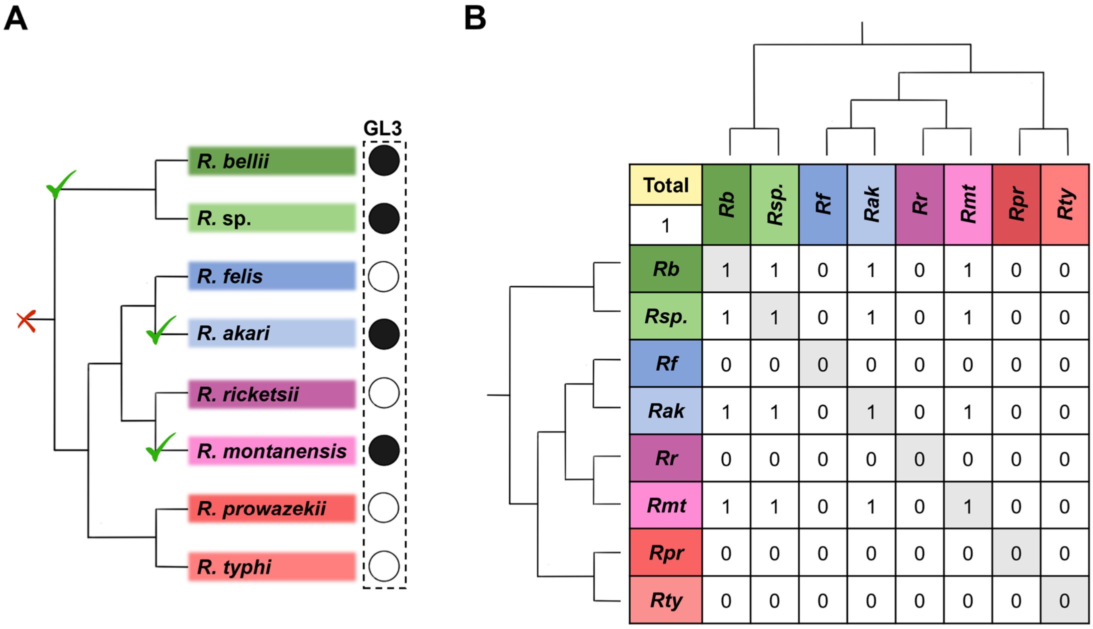

Total 1	Rb	Rsp.	Rf	Rak	Rr	Rmt	Rpr	Rty
Rb	1	1	0	1	0	1	0	0
Rsp.	1	1	0	1	0	1	0	0
Rf	0	0	0	0	0	0	0	0
Rak	1	1	0	1	0	1	0	0
Rr	0	0	0	0	0	0	0	0
Rmt	1	1	0	1	0	1	0	0
Rpr	0	0	0	0	0	0	0	0
Rty	0	0	0	0	0	0	0	0

Figure S1. Example of GL Groupings: **A** is an expand example of a GL3 homologue set with species names included. Closed circles represent the presence of the homologue in the species, while open circles indicate absence. Check marks in the phylogenetic tree indicate instances of gene gain in the phylogeny while the red "x" indicates gene loss or absence. The gain loss pattern shown in this image is one of the two patterns with only 3 gene gain/loss events occurring after the first split in the tree. **B** is an example of the kind of table that appears in Figure 6B and 6C. In this case, the table contains only the example homologue shown in **A.**
